# Epistatic Interaction Between 5-HT1A and Vascular Endothelial Growth Factor Gene Polymorphisms in the Northern Chinese Han Population With Major Depressive Disorder

**DOI:** 10.3389/fpsyt.2019.00218

**Published:** 2019-04-16

**Authors:** Dong Han, Zhengxue Qiao, Dong Qi, Jiarun Yang, Xiuxian Yang, Jingsong Ma, Lin Wang, Xuejia Song, Erying Zhao, Jian Zhang, Yanjie Yang, Xiaohui Qiu

**Affiliations:** Medical Psychology Department of Public Health Institute of Harbin Medical University, Harbin, China

**Keywords:** major depressive disorder, genetic association, 5-HT1A, vascular endothelial growth factor, gene–gene interaction

## Abstract

**Aims:** Serotonin 1A receptor (5-HT1A) and vascular endothelial growth factor (VEGF) are widely expressed in the neurons of the hippocampus and have significant roles in the pathophysiological processes of major depressive disorders (MDDs). The present study was designed to examine 5-HT1A and VEGF gene polymorphisms and whether the gene–gene interaction of 5-HT1A and VEGF gene variants was associated with MDD.

**Methods:** A total of 264 MDD patients and 264 healthy controls were included in the present genetic study. The rs6295, rs1364043, and rs878567 single-nucleotide polymorphisms (SNPs) in the 5-HT1A gene and the rs699947, rs833061, and rs2010963 SNPs in the VEGF gene were selected for genotypic analyses. The generalized multifactor dimensionality reduction method was employed to assess their interactions.

**Results:** The genotype distributions of the two genes’ respective SNPs were significantly different between patients and controls for 5-HT1A rs6295 (*p* = 0.041) and VEGF rs2010963 (*p* = 0.035); however, no significant allelic variation in 5-HT1A (rs6295, rs1364043, and rs878567) and VEGF (rs699947, rs833061, and rs2010963) was found. The interactions between 5-HT1A (rs6295, rs1364043, and rs878567) and VEGF (rs699947, rs833061, and rs2010963) had a cross-validation (CV) consistency of 10/10 and a *p* value of 0.0107, which was considered as the best generalized multifactor dimensionality reduction (GMDR) model.

**Conclusions:** The interactions between 5-HT1A and VEGF gene polymorphisms may play a key role in the development of MDD in the Northern Chinese Han population.

## Introduction

Major depressive disorder (MDD) is one of the most common complex mental disorders, with symptoms that include loss of interest, periodic depression, and thoughts of suicide. Epidemiologic studies have shown that MDD afflicted an estimated 7%–11% of the general population (1, 2); yet, the underlying mechanism of MDD remains unclear. Genetic factors are likely to play a critical role in its etiology with the total contribution of heritability estimated at approximately 40% (3). Results from family, twin, and adoption studies provided further strong evidence for a genetic component to MDD (4); however, there are studies suggesting that single genetic loci offer weak predictive power for the identification of MDD (5). A large body of evidence found that gene–gene interactions are intricately involved in the phenotypic effect of variation in complex psychiatric diseases, particularly when a specific individual genetic variant is present (6, 7). There are numerous studies that suggest that overlaps exist between biological mechanisms underlying MDD (8). Thus, identifying the genetic mechanisms of MDD susceptibility may contribute to a better understanding of the etiological features of MDD (9).

Previous studies of neuroimaging and postmortem MDD patients found that the average volume of the hippocampus declined 9% and that atrophy of existing neurons and neurogenesis may lead to the pathophysiology of MDD (10–12). There is evidence to show that neurogenesis could decrease the levels of MDD and that chronic treatment with antidepressants may facilitate this decrease and also prevent reductions in hippocampal volume (13, 14). The above findings form the basis of the hypothesis that stress and antidepressants can affect hippocampal neurogenesis and are therefore likely have a role in MDD (15–17).

Increasing results of studies demonstrate that dysregulation of neurotrophic factors are associated with MDD (18, 19). Vascular endothelial growth factor (VEGF) and serotonin 1A receptor (5-HT1A) are widely expressed in neurons and have an important role in the pathophysiological processes of MDD (20, 21). Indeed, a previous study demonstrated that activation of the 5-HT1A gene is enough to induce VEGF expression in the neonatal hippocampus (22). The 5-HT1A gene is located on chromosome 5q11.2-q13 and encodes for one of the most abundant serotonin receptors in the brain. Several studies found that the 5-HT1A gene was in both presynaptic and postsynaptic neurons of the hippocampus (23). In addition, an increasing body of literature confirmed that impaired 5-HT1A expression or function was associated with MDD (24, 25). Subsequent studies showed that the rs6295(C-1019G) 5-HT1A promoter polymorphism was associated with MDD and the response to antidepressant therapy (26–28). The VEGF gene is located on chromosome 6p21.3 (29), which has been shown to play a role in hippocampal neurogenesis (30, 31) and in the response to stress. In addition, increasing numbers of studies have identified a role for VEGF in the pathophysiology of MDD and for the neurogenic and behavioral actions of antidepressants (32). So far, whether the 5-HT1A gene variants are able to stimulate VEGF expression is still unclear.

To date, no studies have been initiated to determine if an association between 5-HT1A/VEGF genetic polymorphisms and MDD exists using multilocus analyses in the Northern Chinese Han population. Hence, the aims of this study were to investigate the possible association of 5-HT1A and VEGF gene variants with MDD and to determine the potential susceptibility of gene–gene interactions in this disease.

## Materials and Methods

### Sample

Blood samples were collected from 528 Chinese Han patients with MDD, who were outpatients and inpatients from the Psychiatry Department of the First Affiliated Hospital of Harbin Medical University. All patients underwent the Structured Clinical Interview for the Diagnostic and Statistical Manual of Mental Disorders (SCID, DSM-IV) to confirm the diagnosis of MDD. All the patients were in their first episode. We used the Hamilton Depression Scale Test to assess the severity of patients who had not received antidepression treatment for 2 weeks prior to participation. Patients with other major mental diseases, brain organic mental disorders, and comorbidity for other psychiatric disturbances were excluded. At the same time, 264 age-, education-, career-, and ethnically matched healthy controls were selected from the same hospital for a physical examination. This study was approved by the Ethics Committee of Harbin Medical University. All participants provided written informed consent.

### Genotyping

Genomic DNA was isolated from blood samples using a MagNA Pure DNA Isolator (Roche, Indianapolis, IN, USA). After DNA extraction, PCRs were used to amplify specific regions of the VEGF and 5-HT1A genes, respectively. Single-nucleotide polymorphism (SNP) analyses were performed using the Taqman allelic discrimination assay on a 7900 systems (Applied Biosystem Inc) according to the manufacturer’s instructions.

### Statistical Analysis

SPSS 19.0 software was used for all analyses. The χ^2^ test was used to analyze the between-group differences in gender and distributions of genotypes and alleles between the patient group and the control group. The Student’s *t* test was used to compare continuous variables. The Hardy–Weinberg equilibrium was evaluated for the genotypic distribution of each SNP by using a Pearson goodness-of-fit test in both patients and controls. Haplotype frequencies in MDD patients and controls were estimated by Haploview 4.2 software. Those haplotypes with *p* value need to do permutation tests 1,000 times. A *p* value of less than 0.05 was considered statistically significant.

Generalized multifactor dimensionality reduction (GMDR) analysis was used to assess gene–gene interactions (33) and is an extension of the multifactor dimensionality reduction method. Briefly, we performed 10-fold cross-validation and tested two six-marker interaction models with 1,000 permutations. The best value of maximized cross-validation (CV), balanced accuracy, and the most significant gene–gene interaction model were provided by GMDR analysis. We used interaction graphs to interpret the SNP interactions of the best model (34).

## Results

A total of 528 participants were included in the present genetic study. **Table 1** shows the demographic and characteristics of study participants. No significant difference was found in mean age and sex ratio between patients and controls.

**Table 1:** Characteristics of study participants.

Variable	Case (*n* = 264)	Control (*n* = 264)	χ^2^/*t*	*p* value
Age (mean ± SD)	43.30 ± 13.87	41.82 ± 12.41	1.29	0.20
Gender (males/females)	78/186	72/192	0.00	1.00
HAMD score	31.62 ± 4.84	–	–	–

Haplotype frequencies in patients and controls were estimated by Haploview 4.2 software. The results of haplotype-based analysis are presented in **Table 2**. Strong LD was observed between rs878567 and rs6295 of the 5-HT1A gene (*D*′= 1.0, *r* ^2^ = 0.72), but haplotype-based analysis found that the 5-HT1A gene rs878567-rs6295 haplotype was not associated with MDD (*p* = 0.040, permuted *p* = 0.242).

**Table 2:** Haplotype-based association analysis results.

Gene	Haplotype	MDD (%)	Control (%)	χ^2^	*P*/permuted *P*
5-HT1A	GCC	0.612	0.600	0.18	0.671/1.000
	TTG	0.197	0.215	0.583	0.445/0.993
	TCC	0.119	0.141	1.268	0.260/0.865
	TCG	0.072	0.044	4.207	**0.040/**0.242

MDD, major depressive disorder.

The boldfaced value indicate p < 0.05.

Genotype distributions for 5-HT1A and VEGF gene polymorphisms were analyzed using the Hardy–Weinberg equilibrium. **Table 3** shows the distributions of genotypes and alleles for the six SNPs in patients and controls. The genotype distributions of two SNPs were significantly different between patients and controls for 5-HT1A rs6295 (*p* = 0.041) and VEGF rs2010963 (*p* = 0.035). However, no significant difference in allele distributions of 5-HT1A (rs6295, rs1364043, and rs878567) and VEGF (rs699947, rs833061, and rs2010963) between patients and controls were found.

**Table 3:** Distributions of genotypes and alleles for study participants.

Gene	SNP ID	Sample	Genotype			*P*	Allele		*P*	OR (95% CI)
VEGF	rs699947		AA	AC	CC		A	C		
		Case	11	94	159	0.610	116	412	0.607	1.709 (0.808–1.439)
		Control	16	91	157		123	405		
	rs833061		CC	CT	TT		C	T		
		Case	11	95	158	0.389	117	411	0.558	1.090 (0.818–1.452)
		Control	18	89	157		125	403		
	rs2010963		CC	CG	GG		C	G		
		Case	56	143	65	**0.035**	255	273	0.423	1.104 (0.867–1.406)
		Control	64	114	86		242	286		
5-HT1A	rs6295		CC	CG	GG		C	G		
		Case	130	123	11	**0.041**	383	145	0.486	1.102 (0.838–1.449)
		Control	148	97	19		393	135		
	rs1364043		GG	GT	TT		G	T		
		Case	89	138	37	0.569	316	212	0.900	0.984 (0.769–1.259)
		Control	96	126	42		318	210		
	rs878567		CC	CT	TT		C	T		
		Case	165	92	7	0.093	422	106	0.760	0.955 (0.708–1.287)
		Control	170	78	16		418	110		

SNP, single-nucleotide polymorphism; VEGF, vascular endothelial growth factor; 5-HT1A, serotonin 1A receptor.

The boldfaced values indicate p < 0.05.

In addition, gene–gene interactions were analyzed using GMDR software to assess the impact of combinations of the six SNPs in MDD. *p* values were calculated by permuting the cases and controls 1,000 times. The results of GMDR analysis for each 2-locus to 6-locus multilocus–genotype combination are shown in **Table 4**. The interactions between 5-HT1A (rs6295, rs1364043, and rs878567) and VEGF (rs699947, rs833061, and rs2010963) had a CV consistency of 10/10 and a *p* value of 0.0107, which was considered as the best GMDR model. The results indicated that there were potential gene–gene interactions between 5-HT1A and VEGF in MDD susceptibility.

**Table 4:** Gene–gene interaction analysis by generalized multifactor dimensionality reduction (GMDR).

Locus number	Best combination	Prediction error (%)	Cross-validation	*p* value
2	VEGF (rs2010963), 5-HTR1A (rs6295)	0.4743	6/10	0.6230
3	VEGF (rs2010963), 5-HTR1A (rs6295,rs1364043)	0.403	4/10	0.1719
4	VEGF (rs699947,rs2010963), 5-HTR1A (rs6295,rs1364043)	0.3685	9/10	0.0547
5	VEGF (rs699947,rs2010963), 5-HTR1A (rs6295,rs1364043,rs878567)	0.3493	9/10	**0.0107**
6	VEGF (rs699947,rs833061,rs2010963), 5-HTR1A (rs6295,rs1364043,rs878567)	0.3474	10/10	**0.0107**

The boldfaced values indicate p < 0.05.

**Figure 1:**
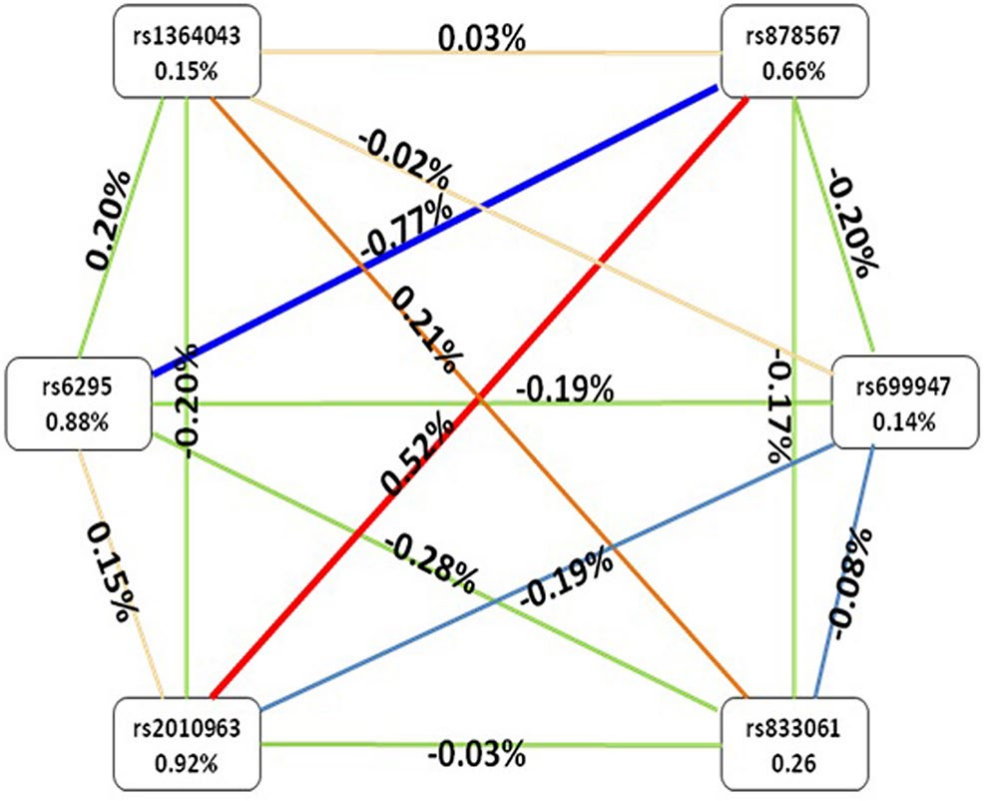
Interaction graphs showed that the percentage at the bottom of each SNPs represented entropy of it, and there are a percentage on line represented the interaction percentage of entropy between two SNPs. The red line represented synergy redundancy interaction and the blue line represented redundancy interaction.

After identifying the impacts of combinations of the six SNPs using GMDR, we used the entropy estimates to produce an interaction graph to interpret the relationship between these six SNPs. As shown in **Figure 1**, a negative interaction effect of rs6295 and rs878567 was found in the 5-HT1A gene with an interaction entropy of −0.77%, while a positive interaction effect of rs878567 was found in the 5-HT1A gene and of rs2010963 in the VEGF gene with an interaction entropy of 0.52. The most independent effect and the least effect were rs2010963 (0.92%) and rs699947 (0.14%), respectively.

## Discussion

In the present study, we explored the impact of the 5-HT1A and VEGF genes and gene–gene interactions on the risk of MDD in the Northern Chinese Han population. Our study investigated not only the main effects but also the joint effects of the 5-HT1A and VEGF genes. The results found that the VEGF gene had a few main effects on MDD, but when combined with the 5-HT1A gene, the interaction effect was definitively associated with MDD.

To date, we have found that the 5-HT1A and VEGF genes are widely expressed in the brain and have varied functions; both were reported to be involved in the antidepressant response. Previous studies also indicated that a decrease in expression of the 5-HT1A or in its function was associated with MDD. Although these studies highlighted the important role of genetic variants in the mechanism of MDD, the identification of the risk gene for MDD needs further investigation. The genotype frequencies of the 5-HT1A rs6295 polymorphism were a risk factor for MDD; however, our analyses show that there was no significant allelic association (*p* = 0.486) between the 5-HT1A rs6295 polymorphism and MDD.

Genetic association studies of 5-HT1A in MDD have produced contradicting results. Wu et al. found that rs6295 (C-1019G) was associated with MDD in the Chinese population (35, 36), while Albert and Lemonde reported that the 5-HT1A rs6295 (C-1019G) polymorphism had an effect on suicide, depression, anxiety, and antidepressant responses (37, 38). Moreover, Illi et al. demonstrated that no association was found between the 5-HT1A gene and MDD or antidepressant responses (39). Yet, Kato et al. reported that the 5-HT1A rs10042486 and rs1364043 polymorphisms were associated with MDD and antidepressant responses (26). A possible explanation for the above findings could be the small sample size employed in these respective studies. Compared with the study by Illi et al. for example, with 106 outpatient MDD patients of Finnish origin, we recruited 264 Chinese MDD samples. This larger sample size likely had a greater power effect than those studies with smaller sample sizes.

Previous studies have shown that a genetic association exists between the VEGF gene and MDD; however, similar to the 5-HT1A, the results have been inconsistent and at times contradictory. Two recent meta-analyses found that VEGF was associated with MDD and response to antidepressants (40, 41). As for the analysis of single loci, Galecki et al.’s study reported that the frequency of the VEGF variant rs2010963(405G/C) increased in MDD patients compared with healthy controls (C-allele and genotype CC are the risk factors for MDD) (42). Several factors may help to explain the contradictory results in the above studies. Although the association between VEGF polymorphisms and MDD was found, ethnic differences might lead to inconsistent results in analyses of allele and genotype frequencies. In addition, MDD is a complex disease that is usually associated with single-gene or gene–gene interactions, and VEGF polymorphisms alone may not predispose MDD; therefore, it is significant to investigate gene–gene interactions among several genes to explain the complex pathogenic mechanisms of MDD.

We used the GMDR method to analyze the combined effects of the 5-HT1A and VEGF genes in MDD. We found that the 5-locus and 6-locus gene–gene interaction models conferred an increased risk of MDD. The interactions between 5-HT1A (rs6295, rs1364043, and rs878567) and VEGF (rs699947, rs833061, and rs2010963) had a CV consistency of 10/10 and a *p* value of 0.0107, which was considered as the best gene–gene interaction model. Of note, stress can exacerbate depressive episodes and down-regulate both VEGF and its major receptors in the brain, including fetal liver kinase-1 (Flk-1). We found that VEGF–Flk-1 signaling played a major role in the clinical effects of antidepressants. A possible mechanism was that the interaction effect that was activated by the 5-HT1A gene in neuronal and endothelial cell generated antidepressant effects (43). Some studies have also found that cAMP-response element binding protein (CREB) and VEGF can be activated by 5-HT1A gene stimulating pathways (extracellular-regulated kinase and protein kinase B) (44, 45). Several limitations of this study still need to be addressed. First, we collected our samples in the Northern Chinese Han population, and it is noteworthy that the positive associations may be due to chance or to a stratification effect. Second, the sample size of this study was relatively small; further studies with a large sample are needed to test our results. Future research should include other population and ethnicities with large sample sizes to explore interaction between these two genes in MDD.

## Conclusions

In conclusion, the current study used single-locus and multilocus analyses to study genetic polymorphisms and gene–gene interactions between the 5-HT1A and VEGF genes in MDD. Our findings suggest that the interaction between the VEGF gene and the 5-HT1A gene may play a key role in the development of MDD.

## Ethics Statement

This study was carried out in accordance with the recommendations of the Ethics Committee of Harbin Medical University with written informed consent from all subjects. All subjects gave written informed consent in accordance with the Declaration of Helsinki. The protocol was approved by the Ethics Committee of Harbin Medical University.

## Author Contributions

YY and XQ designed and conceived the study. DH, ZQ, DQ, JY, JM, and JZ wrote the manuscript and carried out all of the data analyses. XY, LW, XS, and EZ carried out edited manuscript drafts. All authors read and approved the final version of the manuscript.

## Funding

This study was supported by the National Natural Science Foundation of China (81773536 to YY).

## Conflict of Interest Statement

The authors declare that the research was conducted in the absence of any commercial or financial relationships that could be construed as a potential conflict of interest.
